# Acute suppurative cholecystitis complicated by middle hepatic vein thrombosis: a case report

**DOI:** 10.1093/jscr/rjaf1021

**Published:** 2025-12-28

**Authors:** Fan Gao, Zhe Tang, Luting Zhang

**Affiliations:** Department of Hepatobiliary and Pancreatic Surgery, Shangyu People's Hospital of Shaoxing, Shaoxing University, No. 517, Citizen Avenue, Baiguan Street, Shangyu District, Shaoxing City, Zhejiang Province, 312300, China; Department of Hepatobiliary and Pancreatic Surgery, Shangyu People's Hospital of Shaoxing, Shaoxing University, No. 517, Citizen Avenue, Baiguan Street, Shangyu District, Shaoxing City, Zhejiang Province, 312300, China; Department of Hepatobiliary and Pancreatic Surgery, Shangyu People's Hospital of Shaoxing, Shaoxing University, No. 517, Citizen Avenue, Baiguan Street, Shangyu District, Shaoxing City, Zhejiang Province, 312300, China

**Keywords:** acute cholecystitis, hepatic vein thrombosis, gallstone, case report

## Abstract

Hepatic vein thrombosis is a rare and severe complication of acute cholecystitis. Preoperative diagnosis is challenging, and surgical management requires meticulous planning. A 63-year-old asymptomatic male was admitted for elective inguinal hernia repair. Preoperative computed tomography incidentally revealed gallstones with wall thickening and a suspected hepatic vein thrombus. He was transferred for hepatobiliary consultation. Subsequent magnetic resonance imaging confirmed cholecystitis with middle hepatic vein thrombosis. He underwent open cholecystectomy with adhesiolysis and partial colectomy due to dense inflammatory adhesion. Intraoperative frozen section ruled out malignancy. The patient recovered well after a postoperative course of antibiotics and was discharged on Day 19. This case highlights that acute cholecystitis can progress to complicated hepatic vein thrombosis even in asymptomatic patients. Preoperative imaging is critical for diagnosis and surgical planning. Early surgical intervention following oncological principles (R0 resection) is recommended when malignancy cannot be excluded, even if final pathology is benign.

## Introduction

Gallstone disease is common, but its progression to suppurative cholecystitis with hepatic vein thrombosis is exceedingly rare. Such complications pose significant diagnostic and therapeutic challenges. Thrombosis typically arises from Virchow’s triad: stasis, endothelial injury, and hypercoagulability. In cholecystitis, local inflammation can extend to involve the portal venous system or hepatic veins, though reports of middle hepatic vein involvement are sparse [1, 2]. We present a case of asymptomatic acute suppurative cholecystitis complicated by middle hepatic vein thrombosis, preoperatively suspected to be malignancy.

## Case presentation

**Figure 1:**
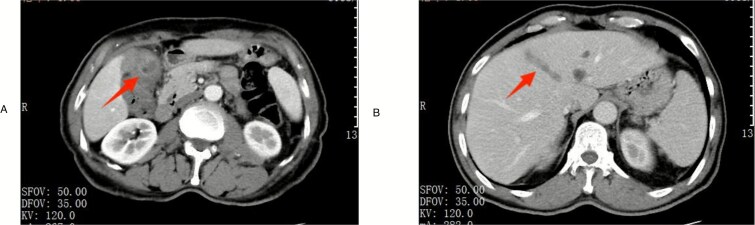
Preoperative imaging. (A) Contrast-enhanced CT scan in the arterial phase demonstrating gallbladder wall thickening with enhancement (arrow). (B) Contrast-enhanced CT in the venous phase showing thrombus formation within the middle hepatic vein (arrowhead).

A 63-year-old male presented for elective right inguinal hernia repair. Preoperative laboratory tests were unremarkable except for elevated C-reactive protein (11.4 mg/L). Abdominal computed tomography (CT) incidentally demonstrated gallstones, significant gallbladder wall thickening (Fig. 1A), pericholecystic stranding, and a filling defect in the middle hepatic vein suggestive of thrombosis (Fig. 1B). Tumor markers (Alpha-fetoprotein [AFP], CA19-9) were within normal limits.

The patient reported a history of gallstones diagnosed in 2020 but had remained entirely asymptomatic, with no episodes of biliary colic. Physical examination revealed no abdominal tenderness or Murphy’s sign.

Given the high suspicion of gallbladder cancer with vascular invasion, the patient was transferred to the hepatobiliary unit. Magnetic resonance imaging and contrast-enhanced CT further supported the diagnosis of cholecystitis with secondary middle hepatic vein thrombophlebitis.

**Figure 2:**
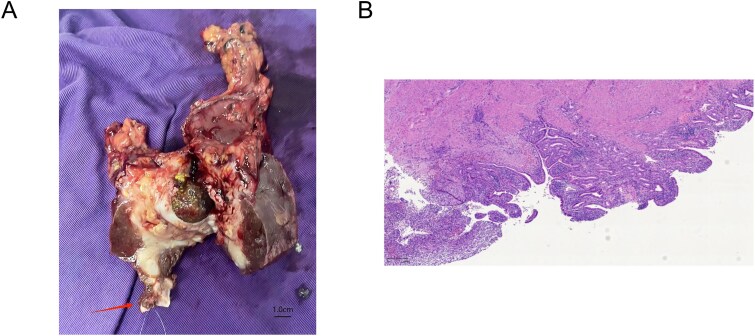
(A) Gross surgical specimen of the gallbladder and adjacent liver tissue, showing the site of thrombus formation in the middle hepatic vein (arrow). (B) Microscopic image showing chronic suppurative cholecystitis (Giemsa stain, ×50).

An open cholecystectomy was performed. Intraoperatively, the gallbladder was severely inflamed and densely adherent to the transverse colon. Adhesiolysis and partial colectomy with primary anastomosis were necessary. A cholecystectomy with a margin of liver tissue was performed adhering to the principle of R0 resection for suspected malignancy. Gross examination of the resected specimen clearly revealed thrombus formation within the middle hepatic vein (Fig. 2A). Intraoperative frozen section pathology confirmed chronic suppurative cholecystitis with no evidence of malignancy.

The patient’s postoperative course was complicated by fever, which resolved with antibiotic therapy. He was advanced to a normal diet and discharged on postoperative Day 19. Final pathology confirmed chronic suppurative cholecystitis and gallstones, with serosal inflammation of the resected colon and liver segments. The thrombus was organized and inflammatory in nature (Fig. 2B).

## Discussion

This case is notable for the development of middle hepatic vein thrombosis, a rare complication of acute cholecystitis, in a completely asymptomatic patient. To our knowledge, only a handful of cases have been reported in the literature describing hepatic vein thrombosis secondary to cholecystitis, with middle hepatic vein involvement being particularly uncommon [3]. This underscores the insidious nature of gallstone disease and the potential for severe complications even without classic symptoms.

The pathogenesis of hepatic vein thrombosis in this context is likely multifactorial. Direct inflammatory extension from the gallbladder fossa to the adjacent hepatic veins can cause endothelial injury, activating the coagulation cascade [4]. Additionally, the systemic inflammatory state induced by suppurative cholecystitis can promote a hypercoagulable state, fulfilling components of Virchow’s triad [1, 5]. The resulting thrombus poses a significant risk of propagation or embolization, including the potential for pulmonary embolism, making timely intervention critical [6].

The preoperative radiographic findings, including significant gallbladder wall thickening and a contiguous filling defect within the middle hepatic vein, were highly suspicious for malignancy, specifically gallbladder cancer with vascular invasion. This differential diagnosis was prioritized due to the imaging characteristics and the known association between gallbladder malignancy and vascular involvement [7, 8]. The normal tumor marker levels (AFP, CA19–9) were reassuring but not sufficient to rule out malignancy definitively, as they can be within normal limits in a subset of gallbladder cancers [7].

Given the high preoperative suspicion for malignancy, the surgical strategy adhered to oncological principles aimed at achieving an R0 resection. This approach is recommended by guidelines when preoperative findings are suspicious for gallbladder cancer, as it ensures complete tumor removal and minimizes the risk of positive margins, which is crucial for prognosis [8]. Intraoperative frozen section analysis was utilized to guide the extent of resection. Although the final pathology confirmed a benign process, the initial surgical strategy was justified to mitigate the significant risk associated with a potential malignancy. This case demonstrates that applying oncological principles in such ambiguous scenarios provides a safety net, ensuring optimal outcomes even when the final diagnosis is benign.

The patient’s postoperative course, though prolonged due to fever managed with antibiotics, was ultimately successful. This outcome highlights the importance of a multidisciplinary approach and meticulous surgical planning in complex hepatobiliary cases.

This case reinforces the updated guidelines advocating for earlier cholecystectomy in patients with gallstones, even if asymptomatic, to prevent such complex complications [9]. The potential for severe, albeit rare, sequelae like hepatic vein thrombosis should be part of the risk–benefit discussion with patients. Patient education and timely referral to specialist care upon discovery of gallstones are crucial components of proactive management.

## Conclusion

Acute cholecystitis can rarely present with hepatic vein thrombosis, mimicking malignancy. A high index of suspicion and detailed preoperative imaging are key to diagnosis. The differential diagnosis should include malignancy, and surgical management should adhere to oncological principles (R0 resection) if malignancy cannot be excluded preoperatively. Intraoperative frozen section can be a valuable adjunct. This case supports the rationale for proactive management of gallstone disease to prevent severe sequelae and underscores the importance of a tailored surgical approach in complex presentations.

## Patient perspective

The patient was satisfied with the treatment outcome and recovery process.

## Approval of the research protocol

This study was conducted in accordance with the declaration of Helsinki. This study was conducted with approval from the Ethics Committee of Shangyu People’s Hospital of Shaoxing.
